# Severity of *Schistosoma haematobium* co-infection with malaria in school-children is potentially modulated by host CD14 gene variants

**DOI:** 10.1186/s13104-023-06479-9

**Published:** 2023-09-08

**Authors:** Mary A. Oboh-Imafidon, Sabrina M. Torbit, Swathi Jacob, Marissa N. Schroeter, Ashley R. Tucker, Olusola Ojurongbe, Bolaji N. Thomas

**Affiliations:** 1 Department of Biomedical Sciences, Rochester Institute of Technology, 153 Lomb Memorial Drive, Rochester, NY 14623 USA; 2 Department of Medical Microbiology and Parasitology, Ladoke Akintola University of Technology, Ogbomosho, Nigeria; 3 Center for Emerging and Re-emerging Infectious Diseases (CERID), Humboldt-Bayer Foundation Research Hub, Ladoke Akintola University of Technology, Ogbomosho, Nigeria

**Keywords:** Schistosomiasis, Co-infection, Malaria, CD14, Innate immunity, Variants, Egg count, Merozoites surface protein

## Abstract

**Objective:**

Schistosomiasis remains a chronic disease of global importance, especially in many rural areas of the world where co-infection with *Plasmodium falciparum* is common. It is critical to decipher the role of single or co-infected disease scenarios on immune system regulation in such individuals and how such co-infections can either ameliorate or complicate immune response and the consequent disease outcome. First, 10 ml of urine samples, collected between 10:00 am and 2:00 pm, was filtered for diagnosis of schistosomiasis, while egg count, indicative of disease severity, was determined by microscopy. Furthermore, genomic DNA samples extracted from dried blood spots collected on filter paper from one hundred and forty-four *Schistosoma haematobium*-infected school-children was tested for *P. falciparum* parasite positivity by an allele-specific nested-PCR analysis of merozoite surface protein (msp)-1 and -2 genes and a real-time PCR assay. In addition, among *P. falciparum* parasite-positive individuals, we carried out a Taqman SNP genotyping assay to extrapolate the effect of host CD14 (-159 C/T; rs2569190) genetic variants on schistosome egg count.

**Results:**

Of the 144 individuals recruited, *P. falciparum* parasite positivity with msp-1 gene were 34%, 43% and 55% for MAD20, RO33 and K1 alleles respectively. Of the co-infected individuals, CD14 genetic variants ranged from 18.8% vs 21.5%, 33.3% vs 44.4%, 9.7% vs 11.8% for single versus schistosome co-infection for the wild type (CC), heterozygous (CT) and mutant (TT) variants respectively. Though the mean egg count for co-infected individuals with CD14 wild type (33.7 eggs per 10 ml of urine) and heterozygote variants (37.5 eggs per 10 ml of urine) were lower than that of schistosome infection alone (52.48 and 48.08 eggs/10 ml of urine respectively), it lacked statistical significance (p-value 0.12 and 0.29), possibly reflecting the benefit of the CD14 activation in schistosome plus malaria co-infection and not schistosome infection alone. In addition, the lower mean egg count in co-infected individuals reveal the benefit of downstream Th1 immune response mitigated by CD14 innate activation that is absent in schistosome infection alone.

## Introduction

Schistosomiasis is one of the most common water-borne disease caused by parasitic trematodes of the genus *Schistosoma*, whose global burden as of 2017, was estimated at 1.4 million disability-adjusted life-years [1]. Globally, 250 million people are affected by this disease, with more than 90% of these people living in sub-Saharan Africa (sSA), and of these individuals, more than half of them are school-aged children. Nigeria, Tanzania, Ghana, Mozambique and the Democratic Republic of Congo accounts for approximately 78 million of the global cases [2–4]. There are six species causing human infection; of these, *S. haematobium* and *S. mansoni* are the most predominant species in many endemic regions [5]. *S. haematobium* is the most common in Nigeria, presenting with disease-associated complications such as hematuria, hydronephrosis and predisposition to the human immunodeficiency virus infection [2, 3, 6–8]. Malaria, on the other hand, remains a disease with significant consequence in many parts of the world, especially among children and pregnant women in sSA and non-endemic travelers [9]. Local residents with some degree of immunity through response to previous exposures, abound in endemic areas [10]. Many developing countries, such as those in sSA, are endemic for both schistosomiasis and malaria [1], along with a myriad of other infectious pathogens, leading to co-infections in the same region and individuals with multiple disease burdens. School aged-children, who are the most vulnerable to both schistosomiasis and malaria, through their involvement with water contact activities, and exposure to infected mosquito bites.

Host genetics and immune response have been shown to be mitigating factors driving infectivity and disease severity among individuals exposed to the same rates of infection [11]. This genetic linkage has been demonstrated to result in varied clinical outcomes. Cluster of Differentiation 14 (CD14), an important pattern recognition receptor for microbial products [12], and localized on chromosome 5q31.1 region, encodes a glycophosphatidylinositol-anchored membrane glycoprotein [13], and is expressed by majority of immune response cells, playing a key role in innate immunity. It exists in two forms; as membrane or soluble form. Membrane CD14 are usually expressed in neutrophils, monocytes, macrophages where it acts during pro-inflammatory response [14, 15]. As a key player in innate immunity, alterations in CD14 expression or polymorphism have been associated with immune response to different diseases and conditions such as autoimmune disease [13], liver disease susceptibility [16], asthma and atopy [17, 18], ischemic heart disease [19], tuberculosis risk [20] and even sickle cell disease [21]. Of recent, we demonstrated its essentiality as a requirement for the evolution of speciation, disease tolerance or susceptibility with bovine trypanosomiasis [22, 23]

The homozygous mutant allele of the CD14 promoter gene polymorphism (rs2569190) has been shown by our group to be associated with susceptibility to disease and regulation of *P. falciparum* malaria parasitemia [24]. Our results demonstrated that CD14 polymorphism potentiates a Th1 differentiation and subsequent interferon-γ response leading to reduced malaria parasitemia. Two other studies, which examined another CD14 locus (rs5744454) reported opposite outcome, with regard to any association between this polymorphism and severe *P. falciparum* infection. While Chakraborty et al. [25] identified a correlation of the wild type allele-CC, with severe malaria, Owade [26] detected no such association. To the best of our knowledge, we have not found any other report that has examined association of this CD14 locus (rs2569190) in malaria and schistosomiasis co-infected individuals.

Beyond *P. falciparum* positivity or severity, along with the high rates of schistosomiasis and malaria endemicity in sSA, it is necessary to delineate how schistosome infection alone or co-infection with malaria modulates immune response and disease outcome. Given the prevalence, severity and impact of *S. haematobium* infection in rural Nigeria, including the potential for co-infection with *P. falciparum*, we set out to demonstrate the role of CD14 genetic variants on disease severity and outcome variables among school-children infected with schistosomiasis alone or co-infected with malaria in Nigeria.

## Materials and methods

### Study setting, design and population

This was a cross-sectional study conducted between January and October, 2015. Study participants were from Ore and Ilie communities in Osun state, Nigeria where schistosomiasis has been reported to be endemic. The lake, harboring the snail intermediate host, is freely accessible for all forms of water contact activities, including recreational use by school children. Inclusion criterion is residency in the study area for more than a year, and school-children must be below the age of 19 years, as previously described [10]. All methods were carried out in accordance with relevant guidelines and regulations or declaration of Helsinki.

### Sample collection and laboratory procedures

## Urine samples

Urine samples, collected between 10:00 AM and 2:00 PM from all consenting individuals or school-children whose parents and guardians had provided consent, were screened for schistosome infection. For those classified as *S. haematobium* positive, an egg count was conducted. To do this, 10 ml of well-mixed urine was aspirated and carefully forced through a filter membrane. The filter was removed, placed on a slide and examined under a 10X objective of a light microscope. The number of eggs on the filter was counted and recorded as the number of eggs per 10 ml urine. From all the collected slides, 10% were randomly selected and re-examined by an independent microscopist for quality control. In addition, demographic data and other correlates of disease (age, sex, packed cell volume-PCV) were collected using a short questionnaire, as previously described [10]. All methods were carried out in accordance with relevant guidelines and regulations or declaration of Helsinki.

### Blood samples

Finger pricked blood was spotted onto filter papers (GE Healthcare Bio Sciences, Piscataway, NJ, USA) from each patient whose urine sample was collected. Genomic DNA was extracted from filter paper blood spot with the Qiagen Blood Mini Kit (Qiagen Inc., Valencia, CA, USA) following manufacturer’s instruction, eluted in 100 μl total vlume and stored at –20 °C until further analysis. All methods were carried out in accordance with relevant guidelines and regulations or declaration of Helsinki.

### Merozoite surface protein 1 and 2 allele genotyping

Primers for the polymorphic regions of interest (msp-1 block 2 and msp-2) was used for a two-step PCR amplification of the extracted genomic DNA from the blood samples, as previously described [20]. For primary PCR reactions, we used 1 μl of genomic DNA template, while 2 μl of the primary PCR product served as template for all nested PCR reactions. All amplification reactions were carried out using the Lucigen EconoTaq Plus Green 2X Master Mix PCR system (Lucigen Corporation, Middleton, WI, USA) in a final volume of 25 μl. Five microliters of amplified PCR products were resolved on 2% ethidium bromide-stained agarose gel and visualized under a UV transilluminator, as described [27].

### Real-time PCR assay for P. falciparum infection

In addition to using the msp-1 and 2 allele specific typing for determining *P. falciparum* parasite diversity, we also utilized the real-time PCR (RT-PCR) assay targeting the *P. falciparum* multi-copy var acidic terminal sequences (varATS) gene, for confirmation purposes [28]. Primers, probe sequence and thermal cycling conditions for RT-PCR validation of *P. falciparum* have been previously described [29]. A cycle threshold (CT) cut-off of ≤ 40 CT was used to score samples as positive for malaria. All methods were carried out in accordance with relevant guidelines and regulations or declaration of Helsinki.

### TaqMan SNP genotyping assay for CD14 gene

To determine the frequency of CD14 genetic variants, we carried out a single nucleotide polymorphism (SNP) assay. In brief, SNP genotyping was performed using Thermo Scientific Taqman SNP genotyping master mix, primers and protocols, as previously described [30]. A total of 10 µl reaction volume was prepared for each assay containing 1 µl of 20X Taqman SNP genotyping assay, 5 µl of 2 × Taqman Mastermix (Thermo Fisher Scientific, Waltham, MA, USA), 1 µl of 20 ng genomic DNA and 3 µl of nuclease-free water. The reactions were set up for SNP genotyping using the CFX Bio-Rad real-time PCR thermocycler (Bio-Rad, Hercules, CA, USA) with previously described conditions. We generated allele calls with the CFX Manager software; discrepant calls were confirmed by comparing the relative fluorescence 1 (RF1) and RF2 reads. All methods were carried out in accordance with relevant guidelines and regulations or declaration of Helsinki.

### Statistical analysis

The genotypic and allelic frequencies of each variant (wild type—CC; heterozygous—CT, and mutant—TT) were estimated as fraction of the total CD14 variants. Independent t-test was carried out to evaluate the association between CD14 genetic variants and *S. haematobium* egg count, age of participants and the packed cell volume (PCV) of individuals with schistosomiasis and malaria infection or schistosomiasis infection alone. Furthermore, we carried out a binary logistic regression model using schistosomiasis-malaria co-infection as outcome variable, and CD14 genetic variants as independent variable, while controlling for age, sex and PCV. In addition, the mean multiplicity of infection was calculated by dividing the total number of alleles detected for msp-1 and msp-2 genes by the total number of samples, as described [31]. We used Student t-test to compare multiplicity of infection between groups (schistosome-infected alone versus schistosome and malaria co-infected). The expected heterozygosity (He, which is a measure of genetic diversity), was employed to assess population structure of parasites [32]. For all analysis, a p value of  ≤ 0.05 was considered to be a statistically significant difference.

## Results and discussion

This study recruited a total of 144 participants, with a mean age of 10.97 years. There were more male (n = 77) than female (n = 67) participants, while the mean egg count of 38.36 eggs per 10 ml of urine was recorded (Table 1). The heterozygous variant (CT) of the CD14 gene was the most predominant in schistosome-malaria co-infected individuals (44.4%) compared to the schistosomiasis alone group (33.3%). This is followed by the CD14 gene wild type allele (CC), which is more prevalent in schistosome and malaria co-infected individuals (21.5%) than in schistosomiasis infected only (18.8%). The least common CD14 gene variant is the mutant (TT) variant, both in those with schistosome and malaria co-infection and schistosomiasis infection alone. Our result is comparable to previous study where the wild type CD14 allele was most prevalent in malaria infected than among uninfected individuals [24]. One possible explanation for this, we surmise, is that continuous exposure to both infections have led individuals residing in this study location to develop immune resistance mechanisms, usually seen in the fixation of beneficial alleles that abrogate clinical symptoms, and even severe conditions. Given this theory, the CD14 gene mutant type allele therefore is posited as neither beneficial nor protective against either infection, causing its low frequency in the population and of minimal effect.

**Table 1:** Demographics of study participants

Parameters	Frequency
Number	144
Mean age (± SD)	10.97 (3.70)
Sex (%)	
Male	77 (53.5)
Female	67 (46.5)
Egg count (per 10 ml of urine)	
Mean count (± SD)	38.36 (53.33)
Range	1–458

*SD* Standard deviation

**Fig. 1:**
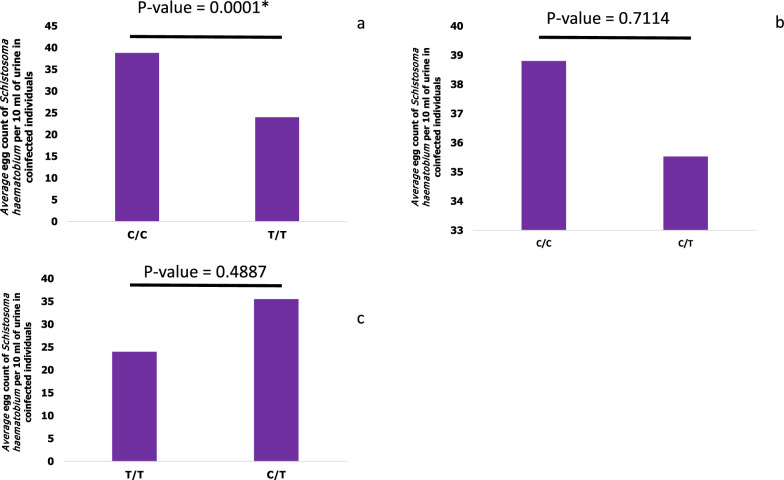
Comparison of mean *Schistosoma haematobium* egg count per 10 ml of urine between CD14 wild type (−159C/C) and mutant (−159 T/T) variant among individuals with schistosomiasis and malaria co-infection and schistosome-infected alone. There was a very high mean egg count and a significant difference between wild type and mutant variants among individuals with schistosome infection alone. The benefit of CD14 expression among individuals with schistosomiasis and malaria co-infection can be seen in the lack of significance

A comparative analysis of *S. haematobium* mean egg count, age of study participants and PCV (indicative of anemia) of the CD14 wild type allele (CC) in schistosomiasis infected individuals with or without malaria revealed an interesting result. Although, urine samples from individuals with schistosomiasis infection alone had a higher mean egg count (52.48 eggs per 10 ml of urine) than schistosomiasis and malaria co-infected individuals (33.70 per 10 ml of urine) (Fig. 1), there was no statistical difference in the mean egg counts between the two categories (p-value = 0.12). However, among individuals with schistosomiasis alone, there was a significant difference in mean egg count between CD14 WT and mutant variants (Fig. 1). Furthermore, no difference was observed in the mean PCV of individuals either in the schistosome-alone or schistosome co-infected with malaria. However, individuals with only schistosomiasis infection were significantly older (mean age: 13 years; t-test 3.51; p-value 0.0009) than those who are co-infected (mean age 9.9 years) (Table 2). This age-associated CD14 wild type (159C > C) allele correlation has also been observed in individuals with tuberculosis [20]. The mutant CD14 allele (159 T > T) showed no protection against higher egg burden in schistosomiasis and malaria co-infected or schistosomiasis infection alone individuals, with mean egg counts essentially even between both groups. Similar results were reported for association of mutant CD14 variant with malaria in southern Nigeria [22]. Taken together, the non-beneficial nature of this allele, its association with higher but insignificant mean egg count, and its low prevalence potentiates immune selection, exerted by the host, leading to a selective sweep and fixation of positively beneficial alleles and exclusion of non-beneficial ones from the population.

**Table 2:** Comparative evaluation of the CD14 genetic variants and infection among single or co-infected individuals

CD14 wild type (−159C/C)					
	Schistosomiasis + malaria (N = 31)	Schistosomiasis only (N = 27)	T-test (Two-tailed)	p-value	CI (95%)
Mean egg count (SEM)	33.70 (5.57)	52.48 (9.99)	1.59	0.12	−18.78 (-42.47 to 4.91)
Mean age in years (range)	9.94 (3–16)	12.96 (5–19)	3.51	0.0009	−3.02 (-4.74 to 1.30)
Mean packed cell volume (range)	37.23 (20–46)	38.70 (29–51)	1.01	0.32194	−1.48 (-4.43 to 1.47)

CD14 wild type (−159C/C) + heterozygotes (−159C/T)					
	Schistosomiasis + malaria (N = 64)	Schistosomiasis only (N = 48)	T-test (Two-tailed)	p-value	CI (95%)
Mean egg count (SEM)	35.73 (8.31)	48.08 (7.62)	1.06	0.2911	−12.35 (-35.43 to 10.73
Mean age in years (Range)	9.88 (3–16)	12.17 (5–20)	3.44	0.0008	−2.29 (-3.61 to 0.97)
Mean packed cell volume (Range)	36.39 (15–50)	37.57 (29–51)	1.11	0.2683	−1.18 (-3.29 to 0.92)

CD14 mutant allele (−159 T/T)					
	Schistosomiasis + malaria (N = 14)	Schistosomiasis only (N = 17)	T-test (Two-tailed)	p-value	CI (95%)
Mean egg count (SEM)	24 (5.52)	21.06 (5.88)	0.36	0.722	2.94 (−13.82 to 19.70)
Mean age in years (range)	10.64 (4–15)	11.94 (4–19)	0.92	0.3645	−1.30 (−4.18 to 1.58)
Mean packed cell volume (range)	37.23 (30–41)	37.35 (29–45)	0.08	0.9403	−0.12 (−3.43 to 3.19)

*SEM* Standard error of mean, *CI* Confidence interval, *NB* The N indicates combined number of individuals expressing wild type (−159C/C), heterozygous (−159C/T) and mutant (−159 T/T) variants in schistosome or schistosome + malaria co-infection state

When the wild type (CC) and heterozygous mutant (CT) alleles were combined and compared between both groups of either schistosome infection alone or in combination with malaria, similar pattern as in the wild type allele was observed. These variants were combined because they provide same level of protection against malaria and schistosome. Additionally, since the number of heterozygous individuals was limited, combining them provide an opportunity to assess their synergistic effect on reducing egg burden and other disease variables.

Although, mean egg count (48.1 egg per 10 ml of urine) and PCV (37.6%) in individuals with schistosomiasis infection alone was higher than in those co-infected with malaria (egg count 35.7; PCV 36.4%), there was no statistical difference (egg count t-test 1.06, p-value 0.2911; packed cell volume t-test 1.11, p-value = 0.2683) between the two groups. Nevertheless, we observed a significant difference in the mean age between schistosomiasis infected versus malaria co-infected group (p-value 0.0008) (Table 2). Binary logistic regression model of schistosome infection status with CD14 genetic variants as a predictor of single schistosome infection or co-infection with malaria demonstrates that those with heterozygous (CT) or mutant (TT) type variant have a 58% odds of being infected (co-efficient of 0.4613 for both heterozygous and mutant schistosomiasis co-infected individuals) with schistosome and malaria concurrently than schistosome infection alone (Table 3). This substantiates the beneficial nature of the heterozygous and/or mutant allele in ameliorating disease severity in co-infected individuals than in schistosome-infection alone.

**Table 3:** Binary logistic regression model of schistosome infection status and CD14 genetic variants

CD14 variant status	Infection status	Coefficient	OR	95% CI	p-value
WT vs heterozygous variant	Schistosomiasis alone	0.0777	1.0807	0.5308–2.2006	0.8304
	Schistosomiasis + malaria	0.4613			
WT vs mutant variant	Schistosomiasis alone	−0.6555	0.5192	0.2227–1.2103	0.1274
	Schistosomiasis + malaria	0.4613			

Although, egg count (48.1 egg/10 ml of urine) and packed cell volume (37.6%) in individuals with schistosomiasis infection alone was higher than in those co-infected with malaria (egg count 35.7; packed cell volume 36.4%) (Fig. 1), there was no statistical difference (egg count t-test 1.06, p-value 0.2911; packed cell volume t-test 1.11, p-value 0.2683) between the two groups. Nevertheless, we observed a significant difference (t-test 3.44, p-value 0.0008) in the mean age between the two groups (Table 1). Although, the egg count in those with schistosomiasis/malaria co-infection in individuals with mutant TT allele was higher (24.0 eggs/10 ml urine) than in those with schistosomiasis alone (21.1 eggs/10 ml urine), no significant difference was observed in the mean egg count (t-test 0.36, p-value 0.722); packed cell volume (t-test 0.08, p-value 0.9403) or mean age (t-test 0.92, p-value 0.3645) between the schistosomiasis co-infected and schistosomiasis infected individuals alone (Table 1).

K1 (16.0%) and 3D7 (17.4%) were the most prevalent alleles in both the msp-1 and msp-2 families respectively. This is followed by RO33 and MAD20 of msp-1 family, while FC27 of msp-2 was the least prevalent. Not surprisingly though, more malaria cases were observed with the real-time PCR assay (86.1%) among the schistosomiasis infected individuals (Table 4). Egg count in either the msp-1 family (K1-37.0 eggs/10 ml of urine; MAD20-35.6 eggs/10 ml of urine; RO33-37.1 eggs/10 ml of urine), msp-2 family (FC27-36.0; 3D7-37.3 egg/10 ml of urine) or those positive by the real-time PCR (37.2 eggs/10 ml of urine) showed no difference. The multiplicity of infection (MOI) for msp-1 was observed to be higher (1.82) than that of msp-2 (1.64) but the heterozygosity between both msp-1 and 2 were similar (He for msp-1 = 0.66; He for msp-2 = 0.63) (Table 4). This is consistent with previous findings in Nigeria [33, 34], Senegal [35], Kenya [36], and Ethiopia [37], where K1 occurs more frequently than other alleles.

**Table 4:** Dynamics of schistosomiasis and *Schistosoma haematobium* egg count in different malaria allelic group

*Pf* allelic genotyping	*Schistosoma* infection N (%)	Mean egg count (± SD	Heterozygosity	MOI
MSP-1				
K1	23 (15.97)	36.97 (55.53)	0.66	1.82
MAD20	8 (5.56)	35.63 (42.46)		
RO33	9 (6.25)	37.06 (57.70)		
MSP-2				
FC27	7 (4.86)	36 (43.29)	0.63	1.64
3D7	25 (17.36)	37.34 (56.25)		
RT-PCR	124 (86.11)	37.21 (55.51)		

Although, both infections co-circulate in endemic regions and among individuals, their interactions are driven specifically by host genetic and immune response and less by pathogen competition. In summary, the immune environment modulated by host CD14 gene in *P. falciparum* parasite positive cases provide some degree of protection that ameliorates disease severity (in terms of egg count) in schistosome-malaria co-infection, especially in younger individuals. This protection is completely abrogated in the schistosome-infected alone.

## Limitations

Our study is limited by its small patient number which may have contributed to the currently observed results. As a single center study, there is the potential that these findings may not be generalizable or representative enough to be applicable other areas with malaria and schistosomiasis co-infection in Africa and elsewhere. Therefore, we recommend that future studies should include a larger sample size covering parts of the continent with different transmission dynamics, and be conducted longitudinally to understand immune dynamics, especially as it relates with schistosomiasis and malaria co-infection.

## Data Availability

All datasets generated or analysed during this study are included in this published article.

## Abbreviations

sSA: Sub-Saharan Africa
CD14: Cluster of differentiation 14
DNA: Deoxyribonucleic acid
MSP1/2: Merozoite surface protein 1 and 2
PCR: Polymerase chain reaction
varATS: Var acidic terminal sequences
RT-PCR: Real-time polymerase chain reaction
SNP: Single nucleotide polymorphism
RF1/2: Relative florescent 1 and 2
MOI: Multiplicity of infection
CI: Confidence interval
OR: Odds ratio
WT: Wild type
